# Formulation and Development of a Water-in-Oil Emulsion-Based Luliconazole Cream: *In Vitro* Characterization and Analytical Method Validation by RP-HPLC

**DOI:** 10.1155/2022/7273840

**Published:** 2022-09-23

**Authors:** Vijay Kumar Panthi, Utsav Nepal

**Affiliations:** ^1^Department of Pharmacy, Tribhuvan University, Sunsari Technical College, Sunsari, Nepal; ^2^Research & Development Department, Royal Sasa Nepal Pharmaceuticals, Chitwan, Nepal; ^3^Research & Development Department, Asian Pharmaceuticals, Rupandehi, Nepal; ^4^Research & Development Department, Corel Pharmaceuticals, Rupandehi, Nepal; ^5^Department of Pharmacy, Kathmandu University, School of Science, Dhulikhel, Nepal; ^6^Quality Control Department, Royal Sasa Nepal Pharmaceuticals, Chitwan, Nepal

## Abstract

Luliconazole (LCZ) is a new antifungal agent containing imidazole moiety which revealed broad-spectrum antifungal activity. The aim of this research was to prepare water-in-oil (w/o) emulsion-based cream formulation of LCZ in addition to the development and validation of an analytical method by reverse-phase high-performance liquid chromatography (RP-HPLC). Cetostearyl alcohol (12.14%), light liquid paraffin (5.00%), white soft paraffin (2.75%), and Tween-80 (1.00%) appeared as the optimized concentration to give better consistency to the cream. Moreover, without adding pH adjusting agents the pH of the optimized formulation (F5) was obtained within the range of human skin pH throughout the stability period. The value of particle size, polydispersity index, and zeta potential was 187.90 ± 2.061 nm, 0.124 ± 0.026, and -10.553 ± 1.349 mV, respectively. In this study, an analytical C18 (4.6 mm × 25 cm), 5 μm column was used for chromatographic separation with a mixture of acetonitrile and water in the proportion of 50 : 50 v/v as the mobile phase at a flow rate of 1.0 mL/min. The calibration curve was obtained linear at 296 nm in the concentration range of 0.08–0.12 mg/mL. Furthermore, the limit of detection (LOD) and limit of quantification (LOQ) were 0.0013 and 0.0042 *µ*g/mL, respectively. In addition, the observed results demonstrated that our developed method was linear (*R*2 = 0.999), precise (%RSD below than 2.0%), and accurate (mean recovery% = 100.18–100.91). The F5 showed no physical changes until 6th month analysis at room temperature and accelerated conditions. Similarly, the assay obtained 101.99% ± 0.27 and 99.89% ± 0.08 at room temperature and accelerated conditions, respectively. Additionally, all validated parameters were obtained within the acceptable limit as well. These findings conclude that both physically and chemically stable w/o cream formulation of LCZ can be formulated and assessed for their stability by applying the authenticated analytical procedure of RP-HPLC.

## 1. Introduction

Luliconazole (LCZ) is a new antifungal agent consisting of imidazole moiety with a broad range of antifungal properties that is structurally associated with its predecessor, lanoconazole [1]. The LCZ has demonstrated its broad range of potentiality against various fungal infections including dermatophytes and onychomycosis [2]. However, the mechanism of its antifungal efficacy is still unclear. The previously published research has reported that this new drug reveals its action by impeding the fungal cytochrome P450; that is, 14-*α* dimethyl enzyme thus inhibits the biosynthesis of ergosterol from lanosterol and then finally hinders the cell wall synthesis within the fungi [3, 4]. Furthermore, to treat several other fungal disorders, the commercial preparation of LCZ cream (1% w/w) was permitted by the United States Food and Drug Administration (USFDA) in the mid of 2013, whereas LCZ cream having the same concentration was authorized for commercialization in Japan and India in 2005 and 2009, respectively [1].

Fungal disorders are considered a serious health issue and an important cause of morbidity. These disorders may be divided into two different categories: superficial and invasive. The superficial type of fungal problems impacts almost 20%–25% of the total population of the world and is related to a disturbance in daily activities, poor quality of life, and expenditure on health care [5]. Similarly, invasive fungal infections are a serious issue that is generally accompanied by the presence of one or more susceptible factors, such as critically ill or immunocompromised patients, and deep or systemic fungal disorders are closely associated with hospitalization and mortality [1]. On the other hand, due to the lower solubility of LCZ, drug permeation hampers through the skin upon topical administration. As a consequence, solubility impedes the LCZ permeation in the lipid phase of the stratum corneum which hinders its dermal availability. Till date, LCZ is commercially available only in cream and lotion formulations. Therefore, various novel deliveries need to be launched in a very urgent manner prior to ameliorating the drug retention and permeation rate from the site of skin delivery. Furthermore, in order to overcome all the problems related to patients' quality of life, physiology, and formulations some research findings are reported in the different previously published literature where many research workers attempted to ameliorate the administration technique of LCZ via fabrication of liposomes and ethosomes, niosomal gel, solid lipid nanoparticle gel, etc. [6, 7]. Regarding the analytical estimation of LCZ, ultraviolet spectroscopy (UV) was also suggested for solubility and stability determination of the drug in the simulated vaginal fluid to evaluate the effect of the vaginal pH and conditions on the drug [8, 9]. Moreover, in pharmacokinetic study the LCZ concentrations were also detected with the help of liquid chromatography-tandem mass spectrometry. Additionally, *in vitro* skin permeability was also previously studied using a UV/visible spectrophotometer [10, 11]. Although cream and solution preparations of LCZ are sufficiently available in the market, still it is very difficult to find a simple and convenient method of high-performance liquid chromatography (HPLC) for the LCZ quantification. The HPLC is a vital analytical instrument in contemporary science, with the feasibility of the maximum number of systems installed and operating globally [12]. Furthermore, modern HPLC provides high resolutions facilitating the quantitative estimation of targeted analytes within the composite matrix by its compatibility with several detectors. To date, there are few nanoformulations of LCZ and their determining analytical method was published. A study done by Manish Kumar et. al prepared LCZ nanocrystals-incorporated hydrogel for the enhancement of antifungal activity and release profile. In this study, *in vitro* dissolution study, drug content uniformity, drug content, etc., were evaluated and *ex vivo* drug permeation rate was assessed using excised dorsal skin of male albino rats. Moreover, in that study, nano-systems exhibited almost 5-fold higher drug solubility, 4-fold enhancement in dissolution study, and better antifungal activity. In addition, the evaluating parameters were analyzed using UV spectrophotometer at a wavelength of 299 nm [13]. Moreover, in another previous research, LCZ nanoemulgel was prepared in order to cure the fungal ailments. In this study, researchers have mentioned that the permeation through the rat skin was remarkably higher with LCZ nanoemulgel in comparison with LCZ gel. Additionally, in case of analytical method, the researchers evaluated entrapment efficiency of LCZ using spectrophotometrically at 299 nm while *ex vivo* drug permeation rate was analyzed by HPLC having mobile phase of ammonium phosphate buffer (0.1 M) and acetonitrile (ACN) at 60: 40 ratio [14]. Moreover, a study done by Tomal Majumder et. al also developed and validated the analytical method of LCZ in marketed cream formulation using RP-HPLC method. In that study, the mobile phase used was water and ACN in the proportion of 60 : 40, respectively, and in addition 2.0 mL/min was flow rate [15], whereas in our study the used solvents were same as them but the composition of mobile phase was 50 : 50 v/v and the flow rate was 1.0 mL/min. Therefore, the main objective of this research was to prepare a stable formulation of water-in-oil (w/o) emulsion-based cream incorporating LCZ in addition to development and validation of simple and precise method of HPLC for quantification LCZ.

## 2. Materials and Methods

### 2.1. Chemicals and Reagents

An optimized formulation of w/o emulsion-based cream containing LCZ was taken from the research and development section of our institution. ACN (HPLC grade) and water (HPLC grade) were procured from Thermo Fisher Scientific India Ltd. Additionally, the working standard and raw material of LCZ were procured from Synergene Active Ingredients, India.

### 2.2. Instrumentation

In this study, Agilent 1260 (infinity II) HPLC system was used for the development and validation of the analytical method. The liquid chromatography (Waldron, Germany) was facilitated with a pump (model: G7116 A), an auto sampler (ALS) (model: G7129 A), and a C18 (250 mm × 4.6 mm) 5 *µ*m packing L1 column (Paisley, UK), and the detector consisted of UV/VIS operated at 296 nm. Agilent OpenLab Software (Version 3.2.0.0) was used for the data processing and evaluation of the obtained results. Furthermore, an analytical weighing balance displaying four digits was utilized (Radwag, model: AS 220.X2, Poland) prior to weighing the analytical reagents, and a sonicator (MRC, model: ACP-150H, India) was applied in order to dissolve the reagents.

### 2.3. Preparation of w/o Emulsion-Based Cream

The overall composition of all creams is mentioned in Table 1. Each preparation was developed by the water-in-oil (w/o) emulsion method. In this study, cetostearyl alcohol, liquid paraffin (light), cetomacrogol 1000, white soft paraffin, and benzoic acid were used in oil phase. Similarly, LCZ, propylene glycol (PG), and Tween-80 (T-80) were used as active phase while sodium lauryl sulfate (SLS), sodium metabisulfite, sodium hydroxide, and purified water were used in the water phase. Briefly, the oil phase containing ingredients of each formulation were accurately and separately weighed and transferred into a 1000 mL beaker; then finally, beaker was kept in a water bath and allowed to heat at 70 ± 5°C with continuous stirring using a glass rod until all excipients were completely melted and dissolved. Accordingly, the water phase consisting of different excipients were separately weighed; then the required quantity of purified water was taken into a 100 mL beaker and placed this beaker in a water bath heated at 70 degree; then the excipients mentioned in each formulation is serially added with continuous stirring until completely dissolved. Moreover, for the preparation of the active phase, take PG into a 1000 mL beaker, keep in the water bath, and heat at 70 ± 5°C; then, LCZ was slowly added with a continuous stirring until clear solution is appeared; then finally, remaining ingredient of each formulation was added in this phase with further stirring. Once all three phases attained an almost similar temperature, the water phase was added slowly into the oil phase and active phase was also added slowly in it and then homogenized at 500 rpm using a high shear homogenizer for 15 minutes to form an emulsion. Finally, after completion of homogenization, the cooling of the emulsion was done at room temperature by placing beaker in a vessel containing cold water, and again further stirring was carried out until a smooth consistency of the cream was obtained.

### 2.4. Characterization of Different LCZ Formulations

#### 2.4.1. Evaluation of pH and Cream Appearance

In this study, measurement of average pH was performed with digital pH meter (Ohaus, Model-Starter 3100C) and before carrying out the measurement, standardization was done using pH 4 and 7 buffer. A 5.0 g sample of o/w cream containing LCZ was weighed and diluted with denoised water containing 95 mL. Then, the pH of each formulation was measured in triplicate. Furthermore, optimized formulation (F5) was observed visually against the light prior to evaluating the optical transparency and the detection of any insoluble drug components or solid particulates. Moreover, shape and surface morphology of F5 was assessed by transmission electron microscopy (TEM). Briefly, to examine the morphological study, the staining of F5 sample was carried out using 2% phosphotungstic acid solution. Then, a drop of the formulation was kept onto a copper grid coated with a carbon film allowed for drying under infrared radiation before viewing under the microscope. The filter paper was used to eliminate the excess matters, and the grid was viewed by TEM (H7600, Hitachi, Tokyo, Japan).

#### 2.4.2. Determination of Zeta Size, Polydispersity Index (PDI), and Zeta Potential

The droplet size, particle size distribution, and zeta potential of optimized formulation were assessed by Zetasizer Nano ZS (Malvern Instruments, Malvern, UK). To assess these parameters, F5 is diluted with purified water (1 : 50), followed by vortex mixing for 2 min. The effect of light scattering was observed at 25°C with a fixed angle of 90°C, and each sample was measured in triplicate.

#### 2.4.3. Viscosity and Spreadability Determination

To determine the viscosity of formulations, Brookfield Viscometer (Brookfield DV-2 + pro) with spindle S64 was used. In this study, all formulations were separately poured into the beaker and then permitted to settle down at 25 ± 1°C for 30 minutes before carrying out the measurement. Subsequently, the spindle was vertically positioned in the center of cream formulations and then carefully examines to ensure that the spindle is not attached to the lower part of the jar and revolved for 10 minutes at a speed of 60 rpm; then, the viscosity value was noted down [16]. Moreover, the spreadability of cream formulations was carried out by accurately weighing 1 g of w/o cream sample and placing within diameter of 1 cm of a premarked circle on a glass plate, and above this, another glass plate was added. A weight of 20 g was allowed for resting on the upper glass plate for 5 min. The area of diameter enhanced due to the spreading of cream was noted, and the mean diameter was determined. The spreading ability of formulation was estimated by applying the following formula:

(1) $$\begin{array}{c} s=\frac{m\cdot l}{t}, \end{array}$$

where *s* denotes spreadability, *m* stands for weight added on the upper slide, *l* defines the length of the upper slide, and *t* denotes the time taken [17].

### 2.5. Analytical Method Development

#### 2.5.1. Chromatographic Conditions

In this research, the mobile phase was prepared by the combined solution of ACN and water (50 : 50 v/v). The filtration of mobile phase was carried out using 0.45 *µ*m membrane filters, and the solution was degassed with sonication for 20 minutes. In this study, an Agilent 1260 HPLC system was used to carry out all the analyses. The dimension of the column was 250 mm × 4.6 mm; 5-*µ*m packing L1 (C18) and the detection were performed by a UV/VIS detector with a detecting wavelength of 296 nm. In addition, flow rate and injection volume were 1.0 mL/min and 20 *µ*L, respectively, and the column was operated at 30°C.

#### 2.5.2. Preparation of Standard Solution

The 10 mg reference standard of LCZ was accurately weighed and transferred into the volumetric flask (50 mL). Afterward, 40 mL of diluent was added and allowed to sonicate for 30 minutes prior to dissolving LCZ; then, volume was made up to the mark with diluents and shaken. Moreover, take 5 mL of standard solution; then, further dilution was done to 10 mL using diluent to obtain final concentration of 0.10 mg/mL, and shaking was done sufficiently; then, 0.20 *µ*m membrane filter was utilized for filtration. In this study, ACN was used as a diluent.

#### 2.5.3. Preparation of Sample Solution

The amount of cream equivalent to 10 mg LCZ (1 gm of cream) was weighed and shifted into the 50 mL of volumetric flask. Furthermore, addition of 40 mL of diluent was done to it and allowed to sonicate for 30 minutes followed by infrequent shaking; then, volume make up was done up to the mark with diluent. Finally, 5 mL of this solution was withdrawn and diluted to 10 mL using diluent prior to get 0.10 mg/mL as the final concentration of test solution then filtered.

### 2.6. Analytical Method Validation

#### 2.6.1. Specificity

Specificity is assumed as the fundamental HPLC parameter that corresponds with the ability of the analytical methods to distinguish between analytes and other excipients in the sample matrix [18]. In this study, specificity was studied by separately injecting the one blank solution, one placebo solution, five replicates of each standard, and sample solution at 100% concentration, and finally retention time and area of both standard and test solution were observed and examined.

#### 2.6.2. Linearity

This parameter deals with the potency to obtain test findings that have a concentration-based correlation with the analyte. In our study, we determined linearity by injecting three solutions of several concentrations (0.08, 0.09, 0.10.0.11, 0.12 mg/mL), and the recovery percentage of each concentration was examined; then, linearity was assessed by visually inspecting the plot area as a function of concentration. Finally, the coefficient correlation (*R*^2^), slope, and intercept were calculated.

#### 2.6.3. Accuracy

This parameter corresponds with the vicinity of the expected value and the obtained findings. In this research, to evaluate assay, the accuracy method was estimated with performing recovery studies at different levels of concentration (80%, 100%, and 120%). The three replicates were injected for each concentration level. Finally, percentage recovery and the value of RSD were estimated for each replicate.

#### 2.6.4. Precision

In this study, to determine the potency, both system and method precision (repeatability) for LCZ were carried out with six measurements of each standard and test samples at 100%. Additionally, by allowing variation in analysts and days, method precision was also determined at the 100% concentration level. The obtained value of %RSD, and all findings are computed in order to find out the results of repeatability study.

#### 2.6.5. Limit of Detection (LOD) and Limit of Quantification (LOQ) Determination

The sensitivity of the assay method of LCZ was examined with six times repeating the calibration curve, and SD intercepts were measured with the help of the following formula prior to examine the value of LOD and LOQ.

(2) $$\begin{array}{c} \text{LOD}=\frac{\left(3.3*S\;D\right)}{\text{Slope}}, \end{array}$$

(3) $$\begin{array}{c} \text{LOQ}=\frac{\left(10*S\;D\right)}{\text{Slope}}, \end{array}$$

where SD = standard deviation of Y intercept of 6 calibration curves and slope = average slope of the 6 calibration curves [19].

#### 2.6.6. Robustness

In accordance with the definition of ICH, robustness corresponds with the ability of the testing method to persist unaltered by minor alternations [20]. In this study, robustness for assay estimation was determined by permitting minor alternations in the flow rate of mobile phase and detection wavelength. The flow rate and detection wavelength were modified by ± 0.2 mL/min and ±2.0 nm, respectively; then finally, assay result including %RSD of the individual sample was evaluated.

#### 2.6.7. Solution Stability

In this research, assay stability of LCZ was performed by evaluating potency of both standard and test samples at 0 h. Furthermore, these samples were also placed in refrigerator and at the ambient temperature (30°C) for 24 h. The five samples of each standard and test solution were examined at 100% concentration for three different condition temperatures; then, the mean of peak area and results of %RSD were determined.

### 2.7. Accelerated Stability Studies

In this research, the stability study of optimized formulation (F5) was performed as per the specifications of the International Conference on Harmonization (ICH). In general, the guidelines recommended by ICH for long term and accelerated storage conditions are 25°C ± 2°C/60% relative humidity (RH) ± 5% RH and 40°C ± 2°C/75% RH ± 5% RH, respectively. If appropriate, an intermediate storage condition (30°C ± 2ºC/65% RH ± 5% RH) is specified according to ICH guidelines [21]. In this study, the formulation filled in a poly-laminated tube was exposed to accelerated stability testing for 6 months as per the ICH norms at a temperature (40 ± 2^o^C) and relative humidity of 75 ± 5%. The samples were analyzed at different durations such as 1st month, 3rd months, and 6th months for the change in pH, color change, and assay. Any unusual changes that appeared in these parameters were recorded. The evaluation of each parameter was performed in triplicate, and both average values were calculated along with the standard deviation.

## 3. Results and Discussion

### 3.1. Preparation, Development, and Optimization of LCZ w/o Formulation

In this study, all five formulations were prepared by the w/o emulsion-based method consisting of mixing with continuous stirring. Each formulation contains the same fill weight (10.00 gm) in a poly-laminated tube. Before carrying out the formulation, the physicochemical properties of LCZ were studied from different literature surveys and all the excipients used in this research were already approved by the USFDA. In trial first (F1), the consistency of the cream was not good and it was quite viscous. Thus, this trial was rejected, and to overcome the previous issue, cetomacrogol 1000 was replaced with white soft paraffin by slightly lowering the concentration in the second formulation (F2) as compared to the amount of cetomacrogol 1000 in trial 1 prior to getting the better consistency of cream by reducing viscosity. In F2, sodium hydroxide was also added in order to get desired pH range compatible with the pH of human body skin. In this formulation, the consistency of the cream was improved in comparison with trial 1; however, the pH of the cream was highly basic. Therefore, this trial was also discarded and in formulation 3 (F3) benzoic acid was added with replacing sodium hydroxide prior to decreasing the basicity of the formulation as well as to getting pH within an acceptable range but in this trial, the cream was observed like hard cake after 24 h of preparation; thereby, this formulation was also rejected. Furthermore, in formulation 4 (F4), both sodium hydroxide and benzoic acid were removed in order to examine the pH range without adding these pH adjusting agents. Interestingly, in this formulation, the cream revealed the appropriate pH (within the range of human skin) but the color was slightly yellowish-white. Thus, in formulation 5 (F5), the concentration of T-80 was reduced in order to assess the impact of this excipient to inhibit the unusual color that appeared in the previous attempt. Finally, in this trial the appearance, consistency, and pH of the cream formulation were achieved better; therefore, this trial was filled and sealed in a laminated tube and finalized for the detailed examination of the formulation. In this study, the overall procedure of formulation and development of LCZ w/o cream is mentioned in section 2.3. Briefly, ingredients containing oil, ingredients consisting water phase, and active phase were separately heated in beaker at 70 ± 5 C. After complete melting and each phase attained the desired temperature, firstly water phase was added slowly to the oil phase with continuous stirring and then the active phase was also added in it with continuous stirring and homogenized at 500 rpm using a high shear homogenizer for 15 minutes to form an emulsion with maintaining the temperature 70 ± 5°C. Furthermore, after mixing three phases the vessel containing cream was allowed to cool by placing in another vessel containing cold water with continuous stirring until better consistency of the cream is achieved. Moreover, in this research, the purpose of ingredient selection was decided as suggested by previously published literature. In brief, cetostearyl alcohol, cetomacrogol-1000, and liquid paraffin were selected because of their emollient, water-absorptive, and emulsifying properties. In addition, liquid paraffin is also used as a solvent and penetration enhancer. Although in this study we were not carried out an animal study prior to examining the permeability rate but purpose of using PG, SLS, and T-80 was to enhance the drug permeation through the skin. Additionally, as the literature suggested, in this study PG was also used as a humectant which pulls water into the skin and helps to keep the skin hydrated [22]. Furthermore, SLS and T-80 belong to the category of anionic and nonionic surfactants, respectively, and the purpose of using these ingredients was to increase the efficacy of topical formulation by enhancing the dug permeation rate as mentioned by previously published studies [23]. Moreover, benzoic acid was applied as a pH adjuster and preservative, whereas sodium metabisulfite and sodium hydroxide were used as an antioxidant and pH balancer, respectively [24, 25].

### 3.2. Evaluation of pH and Cream Appearance

**Figure 1:**
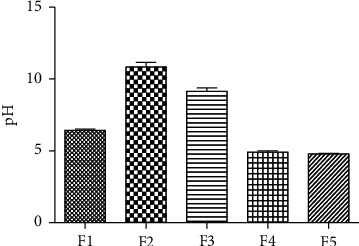
Formulations of LCZ w/o cream; values are expressed as mean ± SD (*n* = 3).

**Figure 2:**
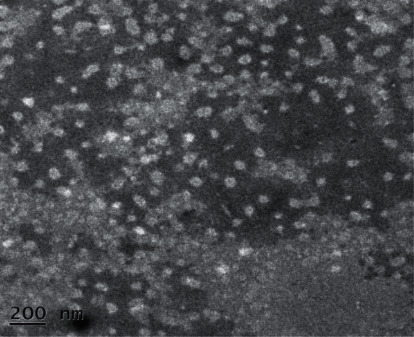
Transmission electron micrograph of optimized w/o (F5) cream.

In this study, F2 and F5 exhibited higher and lower pH values, respectively. The corresponding figures of both these formulations were 10.84 ± 0.31 and 4.79 ± 0.03, respectively. The pH results of all formulations are represented in Figure 1. Among five trials of formulation, only F4 and F5 showed the pH within the range of human skin pH. The pH value of human skin ranges between 4.5 and 6.0 among which 5.5 is considered the average pH of human skin. Thus, the formulations targeted to apply to skin demonstrated the pH within the limit of human skin pH [26]. The F1, F2, F3, and F4 were studied only for pH, spreadability, and viscosity due to improper consistency and higher pH, while F5 was completely studied including the analytical method validation and stability analysis. The creamy consistency of this formulation was found to be better, and the presence of any solid particles was not observed. Moreover, as determined by TEM, the morphology and surface structure of optimized w/o cream formulation also suggested that formulation was spherical (Figure 2).

### 3.3. Particle Size, PDI, and Zeta Potential Assessment

**Figure 3:**
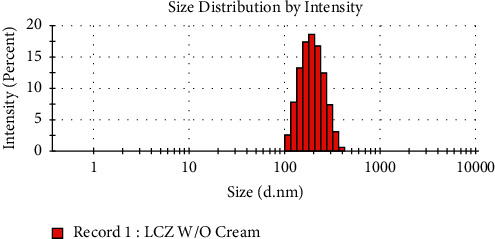
Zeta size of optimized formulation.

**Figure 4:**
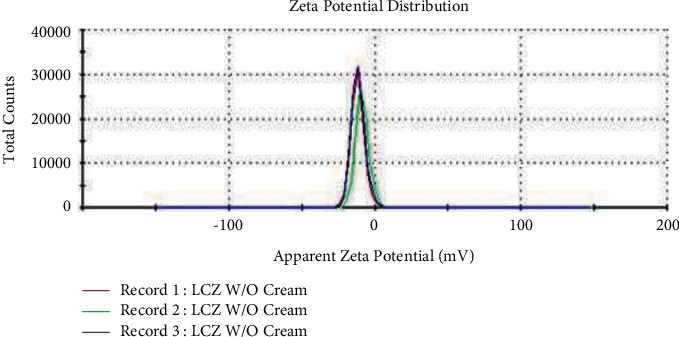
Zeta potential of optimized formulation.

In this study, F5 showed zeta size, PDI, and zeta potential of 187.90 ± 2.061 nm, 0.124 ± 0.026, and-10.553 ± 1.349 mV, respectively. The figure of zeta size and zeta potential is revealed in Figures 3 and 4, respectively. This optimized formulation exhibited homogeneous droplet size distribution. A formulation having value of PDI approximately zero signifies droplet distribution in monodisperse manner. Similarly, PDI having almost one denotes a broad range of droplet distribution while having 0.1 PDI value, exhibiting the distribution of homogeneous droplet size in F5 [27]. Furthermore, this formulation also revealed negative zeta potential value. A study done by Maruno and Rocha-Filho observed remarkable negative value of zeta potentials with nonionic surfactants such as T-80, and this phenomenon is linked with few chemical characteristics of polyoxyethylene chains in the surfactants used [28, 29].

### 3.4. Viscosity and Spreadability Determination

The therapeutic efficacy of topical formulations such as cream and gel mainly depends on the ability of their spreading rate. Therefore, evaluation of spreadability is one of the important aspects of topical delivery [30]. In this study, F1 and F3 demonstrated higher and lower viscosity of 1653 ± 0.27 and 1450 ± 0.29, respectively. Interestingly, the viscosity of F4 and F5 was also almost similar to F2 and F3. Similarly, the spreading rate of each formulation is also approximately very near. Additionally, F3 and F1 showed higher and lower spreadability, respectively. The corresponding figures for F3 and F1 were 6.31 ± 0.12 and 5.66 ± 0.30, respectively. All the data obtained from this study are revealed in Table 2. Moreover, in order to sufficiently permeate the drug via the skin, it is required to exhibit appropriate spreading characteristics, desirable viscosity, and improved skin retention time by topical semisolid preparations. Additionally, it is assumed in general that the viscosity of these formulations is directly interrelated with the proportion of polymer [31, 32].

### 3.5. Method Development and Optimization

In this research, appropriate method was developed for analysis in order to selecting the initial chromatographic conditions of reverse-phase HPLC-UV including stationary phase, mobile phase, detecting wavelength, and technique of sample preparation. Moreover, to achieve this goal, few trials were carried out by modifying the ratio of mobile phase and improving the chromatographic separation conditions on the C18 (250 × 4.6 mm) 5 *µ*m column. The overall findings of method optimization are shown in Table 3.

The various physicochemical characteristics of LCZ were received from the literature study. Meanwhile, various resources reported that LCZ is more soluble in ACN; thereby, we started the preparation of mobile phase employing ACN and water along with their different proportions. In trial 1, the used proportion was 50 : 50 v/v for ACN and water, respectively, and in this trial both HPLC peak and retention time were observed good. Therefore, we decided to accept this trial as a final composition of mobile phase. However, to further evaluate the ACN impact, we decided to attempt other procedures by using this solvent with decreasing and increasing its proportion. In trial 2, we applied ACN and water (40 : 60 v/v) but due to longer retention time this trial was rejected to further use. Similarly, in trial 3, ACN and water were used in the 30 : 70 v/v proportion, respectively, but again we observed the same issue (longer retention time); therefore, this trial was also rejected. Moreover, in trials 4 and 5, we enhanced the ratio of ACN where we used ACN: water in the proportion of 60 : 40 v/v and 70 : 30 v/v, respectively. In these trials, distortion of peak was observed. Thus, these trials were also rejected but in this research, noticeable point is that reducing and increasing the ratio of ACN revealed the higher retention time and peak distortion, respectively. Finally, the mobile phase consisting ACN: water in the ratio of 50 : 50 v/v (trial 1) was accepted. Furthermore, injection volume and flow rate were 20 *µ*L and 1 mL/min, respectively. Additionally, column temperature was operated at 30°C with detecting wavelength at 220 nm using UV detector and these chromatographic conditions were finalized for the detailed study of this research.

#### 3.5.1. Specificity

**Figure 5:**
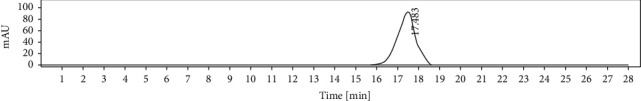
Chromatogram of LCZ standard solution.

**Figure 6:**
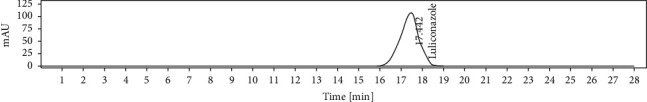
Chromatogram of LCZ sample solution.

In this research, to evaluate this parameter, 20 *µ*L solution of each blank solution, placebo solution, and five samples of both standard and test solution were separately examined at 100% concentration. The chromatograms received from the standard and test solution study are demonstrated in Figures 5and 6, respectively. Moreover, the chromatograms of blank and placebo solution were presented in figures as a supplementary file 1 and 2, respectively. The retention time of the LCZ test sample corresponded with the standard solution and the obtained peak was asymmetrical which further states that there was no interference due to the excipients. All the obtained results finally validated the specificity of the method which is also revealed in Table 4.

#### 3.5.2. Linearity and Range

**Figure 7:**
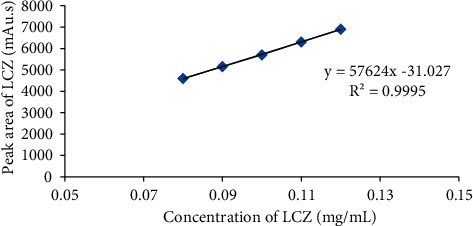
Standard calibration curve of LCZ.

Linearity deals with the potentiality of analytical method to receive test findings that are correspondently dependent on the concentration over a certain range [33]. The average area received from HPLC analysis was organized in accordance with different concentrations of LCZ prior to achieving the calibration curve. This parameter revealed a concentration-dependent interrelationship over the range between 0.08 and 0.12 mg/mL (Figure 7). Moreover, in this concentration range, the *R*^2^ and regression equation of LCZ were 0.999 and *y* = 57624x − 31.02, respectively, revealing a linear interconnection between the concentration of analytes and peak area.

#### 3.5.3. LOD and LOQ

In this research, these parameters were evaluated by estimating the LOD and LOQ of LCZ on the basis of formula shown in Section 2.6.5. The observed value of LOD and LOQ was 0.0013 *µ*g/mL and 0.0041 *µ*g/mL, respectively. Furthermore, the chromatograms of these concentrations for determination of LOD and LOQ were presented as a figure in Supplementary File 3 and 4, respectively.

#### 3.5.4. Accuracy

In this study, an assessment of accuracy was performed in three concentration levels of LCZ such as 80%, 100%, and 120%. Furthermore, each concentration level showed the accuracy in the range of 100.53%–101.21% and the value of % RSD between 0.029% and 0.103% as demonstrated in Table 5. The results of recovery percentage and %RSD were obtained within the acceptable range between 98.0% and 102.0% and not exceeding than 2.0%, respectively, which illustrates the method usefulness for routine drug evaluation.

#### 3.5.5. Precision

In this research, in order to verify whether the method is precise or not both system and method precision were carried out. While determining the system precision, the working system of the chromatographic conditions showed %RSD of retention time and peak area within a suitable range (below than 2.0%). In addition, the number of theoretical plates was observed more than 2000 of each analytical peak. The overall results of system precision are demonstrated in Table 6. Furthermore, in the case of repeatability and method precision evaluation, the %RSD of assay determination was also observed lower than 2.0% and all the obtained data related to the analysis of repeatability and method precision are revealed in Table 7. Moreover, despite varying the days and analysts, the content of LCZ was obtained 99.75%–100.80%. Thus, the results received from all the above studies exhibited that the method is precise.

#### 3.5.6. Robustness

This parameter was assessed by varying the minor alternation of chromatographic conditions including flow rate of mobile phase and wavelength. The results obtained from this study revealed the robustness of the applied method. Although the chromatographic conditions were slightly varied, the findings were observed within the appropriate limit (RSD below than 2.0%). The details of all achieved data are mentioned in Table 8. Additionally, in this study, the assay of LCZ was observed between 99.89% and 100.20%. The variation of chromatographic conditions is not impacted; the results mean that no any noticeable findings were observed.

#### 3.5.7. Solution Stability

In this parameter, the recovery percentage was observed within the suitable range from 99.81% to 99.95% and the value of %RSD was also not more than (NMT) 2.0% which further justified that both standard and sample solutions of LCZ were adequately stable at 30°C and 4°C until 24 h. Moreover, the value of tailing factor and number of theoretical plates were NMT 2.0 and NLT 2000, respectively, which are also observed within the suitable limit as well. The overall findings of the solution stability study are demonstrated in Table 9.

### 3.6. Accelerated Stability Analysis

The optimized formulation (F5) of LCZ cream was assessed both physically and chemically until 6 months under two different environmental conditions. This formulation demonstrated 101.29% ± 0.25 and 102.44 ± 0.31 at room temperature and accelerated stability, respectively. Similarly, until 6 months the assay was observed 99.89% ± 0.08 which verified that the formulated cream was chemically stable. Furthermore, the pH value was also almost similar in 6 months as compared to day 1, and the consistency of the cream was also good throughout the evaluation period. There was no noticeable change in any parameters of the optimized formulation. Therefore, these observations showed that the developed formulation was stable both physically and chemically. The overall results of accelerated stability analysis are mentioned in Table 10.

## 4. Conclusion

In this research, water-in-oil emulsion-based LCZ cream was prepared and a simple and precise RP-HPLC method was developed for analytical assessment and quantification of LCZ in formulated cream. The data obtained from the initial and until the 6th month evaluation of accelerated stability exhibited that formulated cream was both physically and chemically stable. However, still various *in vitro* and *in vivo* study is necessary for the confirmation of its efficacy. Furthermore, validation of this testing method was performed in accordance with the specifications of ICH and verified to be appropriate for the targeted utilization in addition to providing accurate and precise quantitative measurements under slight modification of chromatographic conditions. This research can augment the various research institutions to manufacture the stable formulation of LCZ cream and utilize validated analytical testing procedures to examine the quality of their products.

## Figures and Tables

**Table 1:** Formulation of w/o emulsion-based LCZ cream.

S. no.	Materials (gm/tube)	F1	F2	F3	F4	F5
**1**	Luliconazole	0.100	0.100	0.100	0.100	0.100
**2**	Cetostearyl alcohol	1.254	1.192	1.208	1.224	1.214
**3**	Liquid paraffin (light)	0.450	0.470	0.440	0.480	0.500
**4**	Cetomacrogol 1000	0.3	—	—	—	—
**5**	White soft paraffin	—	0.270	0.272	0.275	0.275
**6**	Sodium hydroxide	—	0.250	—	—	—
**7**	Benzoic acid	—	—	0.150	—	—
**8**	Tween-80	—	—	—	0.25	0.100
**9**	Sodium lauryl sulfate	0.100	0.100	0.100	0.100	0.100
**10**	Sodium metabisulfite	0.010	0.010	0.010	0.010	0.010
**11**	Propylene glycol	1.400	1.400	1.400	1.400	1.400
**12**	Purified water	6.386	6.208	6.320	6.161	6.301
Total weight (gm)	10.000	10.000	10.000	10.000	10.000	10.000

**Table 2:** Spreadability and viscosity of different formulations.

Formulation	Spreadability (mm)	Viscosity (cPs)
**F1**	5.66 ± 0.30	1653 ± 0.27
**F2**	6.15 ± 0.21	1467 ± 0.11
**F3**	6.31 ± 0.12	1450 ± 0.29
**F4**	6.21 ± 0.20	1459 ± 0.13
**F5**	6.14 ± 0.12	1465 ± 0.22

**Table 3:** Results of method optimization.

Column	Mobile phase	Elution mode	Flow rate	Observation	Result
**C18**	ACN : water (50 : 50, v/v)	Isocratic	1.0 mL/min	Good retention time and peak	Accepted
**C18**	ACN : water (40 : 60, v/v)	Isocratic	1.0 mL/min	Longer retention time	Rejected
**C18**	ACN : water (30 : 70, v/v)	Isocratic	1.0 mL/min	Longer retention time	Rejected
**C18**	ACN : water (60 : 40, v/v)	Isocratic	1.0 mL/min	Peak distortion	Rejected
**C18**	ACN : water (70 : 30, v/v)	Isocratic	1.0 mL/min	Peak distortion	Rejected

**Table 4:** Specificity of LCZ.

S. no.	Retention time (minutes)		Area	
	Standard	Sample	Standard	Sample
**1**	17.483	17.442	5686.366	5677.891
**2**	17.503	17.435	5694.328	5652.312
**3**	17.483	17.420	5703.679	5658.390
**4**	17.466	17.405	5642.992	5644.069
**5**	17.457	17.365	5696.565	5629.099
**Average**	17.478	17.413	5684.786	5652.352
**%RSD**	0.102	0.175	0.425	0.319

**Table 5:** Accuracy results of LCZ.

%Spiked level	Replicate number	Peak area	Assay% (w/w)	Recovery%	Mean recovery%	SD	%RSD
**80**	1	4572.268	0.8069	100.86	100.91	0.104	0.103
	2	4571.186	0.8067	100.84			
	3	4580.051	0.8083	101.03			

**100**	1	5704.262	1.0061	100.61	100.56	0.043	0.042
	2	5700.346	1.0054	100.54			
	3	5700.128	1.0053	100.53			

**120**	1	6896.559	1.2146	101.21	100.18	0.030	0.029
	2	6894.528	1.2142	101.18			
	3	6892.162	1.2138	101.15			

**Table 6:** System precision data from the LCZ standard solution of the proposed HPLC method.

Replicate number	RT	Peak area	Number of theoretical plates	Tailing factor
**1**	17.483	5686.366	2885.359	0.900
**2**	17.503	5694.328	2898.566	0.900
**3**	17.483	5703.679	2883.181	0.891
**4**	17.466	5642.992	2860.473	0.889
**5**	17.457	5696.565	2862.859	0.887
**Average**	17.478	5684.786	2878.088	0.893
**%RSD**	0.100	0.4251	-	—

**Table 7:** Results of repeatability and intermediate precision for assay evaluation.

No. of Sample	Sample weight (mg)	Standard area	Sample area	Content of LCZ in cream (% comparing to labeled amount)
*Day1, Analyst 1*				
1	1000.10	5684.624	5708.925	99.97
2	1000.80	5700.509	5700.296	99.75
3	1000.50	5692.440	5710.292	99.95
4	1000.60	5702.616	5704.638	99.84
5	1000.30	5696.776	5708.984	99.95
6	1000.50	5681.942	5706.591	99.89
Average (1–6)				99.89
%RSD (1–6)				0.085

*Day2, Analyst 1*				
7	1000.10	5716.334	5711.201	100.41
8	1000.20	5730.679	5721.259	100.58
9	1000.40	5729.944	5726.216	100.64
10	1000.10	5725.420	5733.249	100.80
11	1000.10	5726.465	5728.945	100.72
12	1000.90	5733.207	5713.012	100.36
Average (7–12)				100.58
%RSD (7–12)				0.171

*Day 2, Analyst 2*				
13	1000.60	5752.034	5711.015	99.81
14	1000.40	5770.969	5710.298	99.82
15	1000.90	5751.785	5716.566	99.88
16	1000.30	5779.596	5721.219	100.02
17	1000.50	5732.576	5708.091	99.77
18	1000.40	5763.467	5706.636	99.75
Average (13–18)				99.84
%RSD (13–18)				0.097

**Table 8:** Robustness data of the proposed HPLC method for LCZ.

Parameters		Avg. std. area (*n* = 5)	% RSD of std. Area	Avg. sample area (n = 6)	%RSD of sample area	%Assay (n = 6)	%RSD of assay
Flow rate	0.8 mL/min	7028.089	0.188	7017.597	0.189	100.49	0.189
	1.2 mL/min (nm)	4699.494	0.074	4676.371	0.271	100.15	0.250

Wavelength	294	5580.059	0.120	5555.64	0.299	100.20	0.304
	298	5668.904	0.177	5626.771	0.378	99.89	0.358

**Table 9:** Robustness data of the applied HPLC method for LCZ.

Parameter	Stability conditions	RT	Avg. peak area	RSD % (peak area)	Tailing factor	Assay%	%RSD (assay)	Number of theoretical plates
Standard solution	0 h	16.97	5629.816	0.159	0.82	—	—	2290.39
	After 24 h at 30°C	17.46	5746.347	0.115	0.80	—	—	2328.47
	After 24 h at refrigerator	17.82	5722.062	0.259	0.81	—	—	2290.36

Sample solution	0 h	17.51	5645.114	0.198	0.83	99.95	0.187	2320.20
	After 24 h at 30°C	17.51	5696.947	0.191	0.82	99.81	0.190	2404.20
	After 24 h at refrigerator	17.28	5674.313	0.406	0.81	99.84	0.409	2266.80

**Table 10:** Stability study parameters for LCZ.

Duration	Temp. (°C)	Humidity (%)	Assay^a^ (%)	Color change	pH^a^	Creamy consistency
1 day	25	NA	101.29 ± 0.25	−	4.75 ± 0.21	+
	40	75	102.44 ± 0.31	−	4.79 ± 0.13	+

1 month	25	NA	101.14 ± 0.18	−	4.91 ± 0.17	+
	40	75	101.55 ± 0.15	−	4.87 ± 0.12	+

3 months	25	NA	102.43 ± 0.23	−	4.85 ± 0.10	+
	40	75	100.56 ± 0.13	−	4.92 ± 0.15	+

6 months	25	NA	101.99 ± 0.27	−	4.77 ± 0.16	+
	40	75	99.89 ± 0.08	−	4.82 ± 0.11	+

^a^Values are expressed as mean ± SD (*n* = 3); +: presence; −: absence; NA : not applicable.

## Data Availability

The data of this study are presented in the form of tables and figures in this article.

## Supplementary Materials

Supplementary Materials Supplementary file 1: chromatogram of blank solution; supplementary file 2: chromatogram of placebo solution; supplementary file 3: chromatogram of LOD determination; and supplementary file 4: chromatogram of LOQ determination with obtained values.
