# Supplementary material for: Formulation and Development of a Water-in-Oil Emulsion-Based Luliconazole Cream: In Vitro Characterization and Analytical Method Validation by RP-HPLC
Source: Int J Anal Chem. 2022 Sep 23;2022:7273840. doi: 10.1155/2022/7273840 (PMC9525796; doi:10.1155/2022/7273840)

Supplementary File 1

FIGURE: Chromatogram of blank solution.


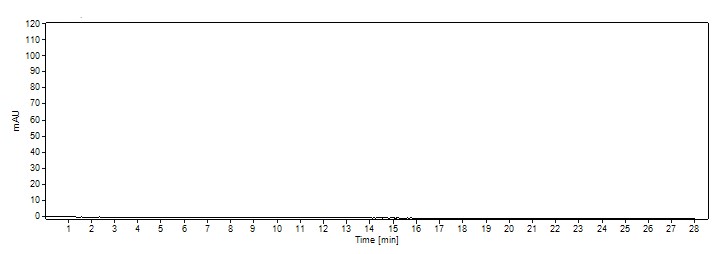


Supplementary File 2

FIGURE: Chromatogram of placebo solution.


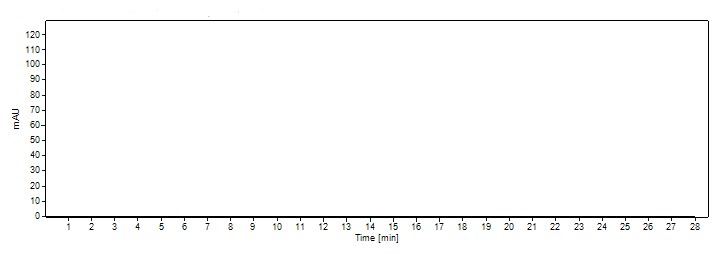


Supplementary File 3

FIGURE: Chromatogram of LOD determination.


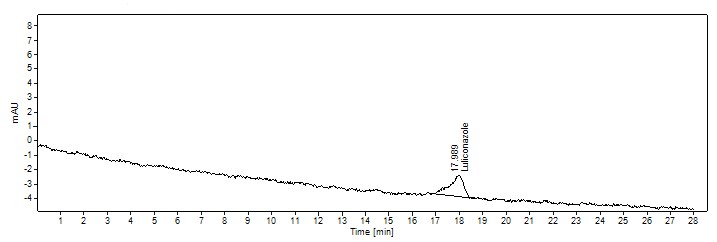


Supplementary File 4

FIGURE: Chromatogram of LOQ determination with obtained values.


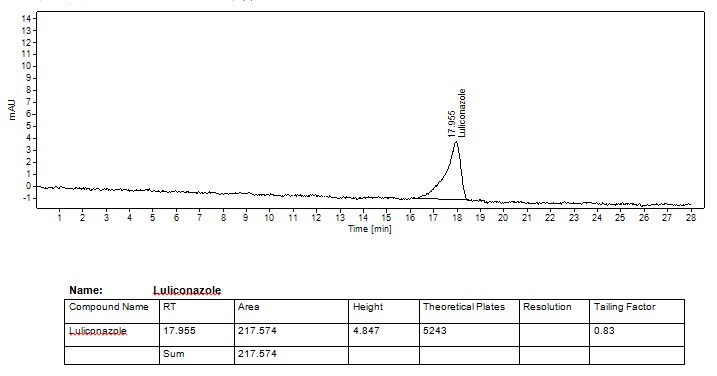

Supplement: Supplementary Materials — Supplementary file 1: chromatogram of blank solution; supplementary file 2: chromatogram of placebo solution; supplementary file 3: chromatogram of LOD determination; and supplementary file 4: chromatogram of LOQ determination with obtained values. [file 7273840.f1.docx]
